# Phylogenetic evidence from freshwater crayfishes that cave adaptation is not an evolutionary dead‐end

**DOI:** 10.1111/evo.13326

**Published:** 2017-09-20

**Authors:** David B. Stern, Jesse Breinholt, Carlos Pedraza‐Lara, Marilú López‐Mejía, Christopher L. Owen, Heather Bracken‐Grissom, James W. Fetzner, Keith A. Crandall

**Affiliations:** ^1^ Computational Biology Institute The George Washington University Ashburn Virginia 20147; ^2^ Department of Biology University of Florida Gainesville Florida; ^3^ Licenciatura en Ciencia Forense, Facultad de Medicina Universidad Nacional Autónoma de México México; ^4^ Lab. Biología Evolutiva y Genética de Poblaciones Universidad de Quintana Roo Cozumel México; ^5^ Department of Biological Sciences Florida International University North Miami Florida 33181; ^6^ Section of Invertebrate Zoology Carnegie Museum of Natural History Pittsburgh Pennsylvania 15213‐4080; ^7^ Department of Invertebrate Zoology, U.S. National Museum of Natural History Smithsonian Institution Washington District of Columbia 20013

**Keywords:** Caves, crayfish, diversification, extinction, habitat, range size, synthesis

## Abstract

Caves are perceived as isolated, extreme habitats with a uniquely specialized biota, which long ago led to the idea that caves are “evolutionary dead‐ends.” This implies that cave‐adapted taxa may be doomed for extinction before they can diversify or transition to a more stable state. However, this hypothesis has not been explicitly tested in a phylogenetic framework with multiple independently evolved cave‐dwelling groups. Here, we use the freshwater crayfish, a group with dozens of cave‐dwelling species in multiple lineages, as a system to test this hypothesis. We consider historical patterns of lineage diversification and habitat transition as well as current patterns of geographic range size. We find that while cave‐dwelling lineages have small relative range sizes and rarely transition back to the surface, they exhibit remarkably similar diversification patterns to those of other habitat types and appear to be able to maintain a diversity of lineages through time. This suggests that cave adaptation is not a “dead‐end” for freshwater crayfish, which has positive implications for our understanding of biodiversity and conservation in cave habitats.

Caves and other subterranean habitats have long been hypothesized as “evolutionary dead‐ends” due to the perceived extreme nature of cave‐dwelling organisms’ morphologies and the high degree of phenotypic and ecological specialization observed in cave inhabitants (Poulson and White 1969; Barr and Holsinger 1985; Culver and Pipan 2009). The most conspicuous features of cave‐adapted taxa are the nearly ubiquitous loss of complex traits such as vision and pigmentation, presumably in response to evolution in a unique aphotic environment (Culver 1982; Culver et al. 1995). Loss of these traits would potentially disadvantage troglobionts (obligate cave‐dwellers) in surface habitats compared to their generalized epigean (surface) counterparts and the reevolution of these traits seems improbable once they are lost (Simpson 1955; Porter and Crandall 2003). As cave‐dwelling species become specialized to the relatively stable environment of subterranean habitats, it creates a situation in which these species are particularly susceptible to environmental perturbations through evolutionary time. A considerable percentage of troglobitic species are geographically isolated and numerically rare (Culver and Pipan 2009), potentially limiting genetic diversity and increasing the potential for local extinctions (Culver 1982; Caccone et al. 1986; Strecker et al. 2003). These factors have led to the idea that once a lineage becomes an obligate cave‐dweller, it may be doomed for extinction before it can diversify or transition to a more evolutionarily stable state, that is, one that can maintain lineages through time.

The degree to which troglobitism can be considered an evolutionary dead‐end depends both on the irreversibility of cave specialization and the ability to maintain lineages through time once adapted to cave environments. It is generally accepted that there is an interplay among dispersal ability, geographic range size, speciation, and extinction that influences a group's evolutionary success (Bilton et al. 2001; Bohonak and Jenkins 2003; Kisel and Barraclough 2010; Sukumaran et al. 2015). Given the typically “patchy” nature of caves and other freshwater ecosystems, the relationship between dispersal ability and speciation rate can be described by an intermediate dispersal model (Diamond et al. 1976). In this model, the relationship between speciation and dispersal forms a curve where the highest speciation rates result from a medium level of dispersal ability that produces a “patchy” distribution in space (Claramunt et al. 2012). Dispersal is somewhat rare, but occurs often enough to allow for occasional range expansion and facilitates genetic isolation. Low dispersal ability results in species with high levels of endemism and little population structure. High dispersal ability results in large geographic range sizes, but also low population structure as individuals are able to explore a high percentage of the range (Claramunt et al. 2012). Lineage diversification may be limited in caves due to restricted dispersal capabilities and high levels of endemism. Even when dispersal is possible (either via washouts or subsurface connections), caves are a relatively homogeneous habitat both spatially and temporally, limiting the number of available niches. As with other ecologically specialized taxa, troglobites are hypothesized to be particularly susceptible to environmental variability, which would increase extinction probability, and cave fauna are frequently in consideration for conservation actions (Culver and Pipan 2009). Therefore, if caves are evolutionary dead‐ends, we would expect cave‐adapted lineages to have relatively small geographic range sizes, low or zero transition rates out of caves, elevated extinction rates relative to speciation and/or transition, and a decreased net diversification rate relative to surface species.

Recent phylogenetic studies have tested and challenged the assumptions of specialization (including specialization to caves) resulting in an evolutionary dead‐end. For example, Prendini et al. (2010) found that epigean scorpions have evolved from cave‐dwelling ancestors multiple times suggesting that cave adaptation is reversible, and while Pyrenean cave beetles have evolved to live in caves only once, they can persist and diversify with success (Cieslak et al. 2014). Day et al. (2016) found mixed support for the evolutionary dead‐end hypothesis for specialists across a set of 10 phylogenies. In general, however, these studies focus on groups with either a small number of specialized taxa or with very few replicated events of specialist evolution.

With approximately 45 described troglobitic species and subspecies (Crandall and De Grave, 2017), the freshwater crayfish are an excellent group in which to test the evolutionary dead‐end hypothesis of cave‐dwelling organisms. Multiple origins of cave adaptation have been hypothesized for crayfish based on morphology and geography (Hobbs et al. 1977) providing power to detect biologically meaningful patterns. Taxonomically, cave crayfish fall into four genera, all of which belong to the largest of the five crayfish families, *Cambaridae* (Hobbs and Barr 1960). These generic placements were made according to genital morphology, geography, and other typical characters used in crayfish taxonomy, suggesting independent origins of cave adaptation. Cave crayfish are known from multiple geographic regions in the United States, Cuba, and Mexico, including the Florida Lime Sinks, the Ozark Plateau, the Cumberland Plateau, the Interior Lowlands, the Greenbriar Valley, and the Sierra Madre Oriental. However, without a complete phylogeny, it is difficult to determine the number of transitions to and from cave life, especially considering the high degree of convergent morphology found in troglobionts. Crayfish species also exhibit other “specialized” habitat preferences (burrows, lentic waters, and lotic waters) which can be compared to caves to test if cave specialization is significantly different from other types of habitat specialization. Here, we estimate the most complete molecular phylogeny of the freshwater crayfish to date and employ phylogenetic comparative methods to test the evolutionary dead‐end hypothesis in cave lineages, considering both historical and current patterns. We test if cave‐adapted taxa have small geographic range sizes compared to surface lineages and other habitat specialists, and test if cave adaptation is an irreversible state with decreased lineage diversification rates relative to surface and other specialized lineages. Taken together, these multiple lines of evidence will support or refute the evolutionary dead‐end hypothesis for cave habitats.

## Materials and Methods

### PHYLOGENY ESTIMATION

Sequences were obtained for three mitochondrial (16S, 12S, COI) and three nuclear (18S, 28S, Histone H3) gene regions for 466 described crayfish species and subspecies, representing 70% of the described diversity (Table S1) (Crandall and De Grave 2017). These genes have proven useful for resolving relationships among crayfish and other decapods (Toon et al. 2009; Bracken‐Grissom et al. 2014). Of the 1565 total sequences from 466 taxa used in the phylogeny estimate, 411 from 119 taxa were new to this study. The full molecular dataset contained 450 16S sequences, 289 12S sequences, 352 COI sequences, 86 18S sequences, 247 28S sequences, and 141 Histone H3 sequences. Protein coding sequences (COI, H3) were aligned by translating to amino acid sequences and back translating to nucleotides in Translator X (Abascal et al. 2010). Protein coding sequences that contained stop codons were removed to eliminate potential nuclear mitochondrial genes (Song et al. 2008; Buhay 2009). The rRNA genes were aligned with PASTA version 1.6 (Mirarab et al. 2015) using default alignment, merging, and tree searching algorithms suggested by the program. We used the greedy algorithm in PartitionFinder version 1.1.1 (Lanfear et al. 2012) to select an optimal partitioning scheme and best fitting models of molecular evolution for each partition using the Bayesian information criterion (BIC). Codon positions for protein coding genes and full rRNA genes were used as a priori data subsets.

Bootstrap phylogenies (*N* = 1000) were estimated from the concatenated alignment using RAxML version 8.2.13 (Stamatakis 2014) with the optimal partitioning scheme suggested by PartitionFinder. Option “‐f d” was used to search for the maximum likelihood phylogeny from 200 randomized stepwise addition order parsimony trees under the GTRCAT model, with the best scoring tree optimized under the GTRGAMMA model with four rate categories.

The maximum likelihood phylogeny and all bootstrap phylogenies were calibrated to absolute time using penalized likelihood in the program treePL (Smith and O'Meara 2012) with an optimal smoothing parameter chosen using a cross‐validation procedure on the maximum likelihood phylogeny. Eight fossil calibration points were used following the placements and justifications found in Bracken‐Grissom et al. (2014). The maximum age of the root of the tree was set to 262 Ma to reflect an estimated split of the two crayfish superfamilies (Astacoidea and Parastacoidea) (Bracken‐Grissom et al. 2014). The lower bounds of fossil age estimates were used as minimum estimates for their assigned clades.

Although our molecular dataset sought to maximize coverage and compatibility with existing sampling, we used a phylogenetic synthesis approach, rather than either molecular sampling or taxonomy alone, to take advantage of all recent phylogenetic studies and taxonomic updates to the freshwater crayfishes. We also used our synthesis tree (our newly estimated phylogeny + phylogenies from the literature + taxonomy) to assess data coverage and sampling (Hinchliff et al. 2015). The synthesis approach allows us to construct a more complete picture of phylogeny and taxonomy, using studies with different sampling schemes (in terms of taxa and genes), which often result in different topologies. This approach takes taxonomy as a backbone phylogeny and introduces bifurcations to the tree using existing phylogenetic studies, resolving conflicts among these inputs with user‐defined rankings. We combined 19 published crayfish phylogenies (as referenced in Owen et al. 2015) and the maximum likelihood estimate with Open Tree taxonomy ott2.10 (Hinchliff et al. 2015). These trees were merged and assembled into a synthesis tree using the *propinquity* pipeline (Redelings and Holder 2017). Taxa not represented in the published phylogenies or in the maximum likelihood estimate are represented by taxonomy in the synthetic tree, giving us a phylogeny‐informed understanding of data distribution and a complete phylogenetic hypothesis for the freshwater crayfishes.

### GEOGRAPHIC RANGE SIZE, HABITAT CORRELATION

We analyzed species’ current geographic range sizes to test if the contemporary distributions of cave lineages are smaller than those of other specialized or generalized surface lineages, a predicted consequence of the dead‐end model. Dispersal ability is generally assumed to have a relationship with geographic range size, despite the many exceptions and other contributing factors to the range size of a species (Lester et al. 2007). Nevertheless, both dispersal ability and geographic range size influence speciation and extinction dynamics (Rosenzweig 1995; Birand et al. 2012). Extinction probability increases to 1 as range size tends to 0 (Jones et al. 2003) and range size is one of the most commonly used predictors of extinction risk (Foote et al. 2008; Harnik et al. 2012). Conversely, speciation probability should increase as geographic range size increases due to the increased probability of vicariance or isolation by distance yielding population fragmentation (Birand et al. 2012).

Range maps in the form of ESRI Shapefiles for 540 crayfish species were obtained from the IUCN Red List database (IUCN 2015) and converted into spatial polygons in the R packages *letsR* (Vilela and Villalobos 2015). Native geographic range sizes in square meters were calculated for each species from the spatial polygons. If multiple polygons were present for a given species, range sizes for each polygon were summed to obtain a total range size estimate for each species. Range sizes were converted to square kilometers and log_10_‐transformed before all analyses.

We used Felsenstein's threshold model to test for a correlation between rates of change in habitat‐preference and geographic range size, thereby testing if cave‐adapted species and other specialized species have significantly smaller geographic range sizes than nonspecialized species (Felsenstein 2012). The threshold model assumes that a binary trait has an underlying continuous trait called “liability” that controls the state of the binary trait. This facilitates estimation of the correlation between a discrete binary and a continuous trait. The maximum likelihood phylogeny was trimmed to the 386 tips with range maps in the IUCN Red List database (Table S2). Habitat preference was coded as either exclusively one state (i.e., cave = 0) or any other state or combination thereof (i.e., surface = 1). Habitat assignments were made using data from the IUCN Red List (IUCN 2015) and crayfish taxonomy web browser (Fetzner 2005). Species were assigned to one or more of the following categories: lentic (still) water, lotic (flowing) water, caves, or burrows. Primary (obligate) burrowers were coded as inhabiting burrows exclusively, whereas secondary (seasonal) burrowers were coded as inhabiting burrows and another habitat state (lentic, lotic, or both) (Table S2). Five tests were performed for each of the four “specialist” habitat types and one “generalist” type using the R package *phytools* (Revell 2012). A Markov chain Monti Carlo (MCMC) chain was run under default priors for 30 million generations for each test, sampling every 3000 generations. The first 25% of samples were discarded as burn‐in prior to summarizing parameter estimates and effective sample size (ESS) values for the correlation coefficient, *r*, were checked (>200) to assess convergence. Correlations were assumed to be significant if the 95% highest posterior density (HPD) interval of the correlation coefficient, *r*, did not contain 0, because the power of the analysis can reliably reveal only the sign of the correlation (Felsenstein 2012).

### DIVERSIFICATION AND TRANSITION RATES

#### Parameter estimation and hypothesis testing

The evolutionary dead‐end model predicts that cave adaptation is an irreversible state leading to increased extinction rates and decreased speciation rates. We used the binary‐state speciation and extinction (BiSSE) and geographic‐state speciation and extinction (GeoSSE) models to estimate diversification and transition rates, and test this prediction of the evolutionary dead‐end hypothesis. GeoSSE was originally intended for use with discrete geographical areas, but is applicable to analyses of habitat preference due to the similar processes involved in range and habitat evolution (Goldberg et al. 2011). For GeoSSE, taxa were coded as obligate cave‐dwellers (A), surface‐dwellers (B), or both (AB). Taxa were only coded as AB if they are found both in surface and cave habitats, and can survive and reproduce in caves (troglophiles). For BiSSE, taxa were coded as obligate cave‐dwellers (0) or not obligate cave‐dwellers (1) to capture the dynamics of specialist species. Incomplete sampling was accounted for by specifying the percentage sampled in each state, estimated from the synthetic tree, and subspecies were trimmed to one per species to avoid conflating species‐ and population‐level processes.

To avoid making process‐based inferences based on point estimates of parameters and model fits, which have been shown to be problematic and potentially misleading (Goldberg et al. 2011; Rabosky and Goldberg 2015; Beaulieu and O'Meara 2016), we took a Bayesian approach to testing these predictions based on MCMC samples of the full (unconstrained) models. For each of 1000 bootstrap phylogenies, an MCMC chain with slice sampling (Neal 2003) was run for 5000 generations using a broad exponential prior distribution with a mean of 0.5 on all parameters of the BiSSE and GeoSSE models in the R package *diversitree* (FitzJohn 2012). The first 10% of samples from each run were discarded as burn‐in. Posterior probabilities of different predictions made by the evolutionary dead‐end hypothesis were assessed by calculating the percentage of MCMC samples that met each condition (e.g., the extinction rate is greater than the speciation rate). We assessed support for an irreversible model by constraining the transition rate out of caves to 0 and enforcing the root state to be in the “surface” state (Goldberg and Igic 2008). Reversible and irreversible models were compared using the Bayes factor (Kass and Raftery 1995) with marginal likelihoods estimated by taking the harmonic mean of the likelihood of the MCMC chains. All analyses were run on two sets of 1000 bootstrap trees: one set containing the full crayfish phylogeny and the second containing the largest pruned subtrees that maximized the percentage of cave species to reduce potential biases associated with an uneven tip‐state distribution (Gamisch 2016). This subtree was identified using the synthetic phylogeny and happened to correspond to the *Cambaridae* family, which contains all described cave‐adapted species.

To test if diversification rates in cave lineages are significantly different from other specialized habitat affinities, we repeated the above procedure for estimating BiSSE and GeoSSE parameters, categorizing taxa into three additional crayfish habitat types, as above. Taxa occurring in more than one habitat were coded as “AB” in GeoSSE and as “state 1” in BiSSE. To facilitate comparisons across separate analyses for each habitat type, we analyzed relative net diversification rates ([*s_A_* − *x_A_*]/[*s_B_* − *x_B_*]).

#### SSE model adequacy

To test the adequacy (objective fit) of a state‐dependent diversification model, we took two approaches. The first was a posterior‐predictive approach in which we simulated phylogenies using parameter estimates from the model and compared the simulated data to the empirical trees. If the model adequately describes the data, phylogenies simulated under the model should be compatible with the observed data (Pennell et al. 2015). First, we tested whether data simulated under estimated model parameters produced the same tip state frequency as the empirical phylogenies. For each sampling scheme and model, we used *diversitree* (FitzJohn 2012) to simulate 1000 phylogenies of the same size as the total number of taxa with coded habitat data. Each simulation was run using parameters from a random sample from the MCMC chain. For each set of trees, we calculated the number of taxa surviving to the present in each state and compared this to the observed state distribution with a chi‐squared test. We also tested if phylogenies simulated using estimated BiSSE and GeoSSE parameters would produce the same phylogenetic signal of habitat states as the empirical data. To keep the prevalence of each state constant, we simulated 1000 trees for each sampling scheme and model using backward simulation in *phylometrics* (Hua and Bromham 2016), using the estimated parameters from 1000 MCMC samples for each model. We compared the phylogenetic signal of habitat states in the simulated data and bootstrap phylogenies using the sum of sister clade differences (SSCD) (Fritz and Purvis 2010) calculated in the R package *phylometrics* (Hua and Bromham 2016). We tested if the mean of the simulated distribution of SSCD was significantly different from the observed distribution using a *t*‐test and calculated the proportion of SSCD values in the bootstrap phylogenies that fell within the 95th and 50th percentiles of simulated SSCD values.

The second approach in testing the adequacy of state‐dependent models in general was to fit a set of hidden‐state speciation and extinction (HiSSE) models to the data to test if an unobserved character in cave lineages might influence diversification dynamics and if character‐independent models fit the data better than the state‐dependent models (i.e., the diversification process varies through the tree, but not according to any particular modeled trait) (Beaulieu and O'Meara 2016). HiSSE allows each state of a binary trait to contain an unobserved “hidden” state with diversification rate parameters that may be different from the observed states. This also facilitates the construction of “character‐independent” diversification models with equal complexity to “character‐dependent” models. When used in a model selection context, this has been shown to reduce the propensity to select state‐dependent models (Beaulieu and O'Meara 2016). As the implementation of these models in the R package *hisse* does not currently support MCMC sampling, we do not extensively draw conclusions based on point parameter estimates given the complex likelihood surfaces of SSE models (Goldberg et al. 2011; Beaulieu and O'Meara 2016). We fit six HiSSE models using the R package *hisse* (Beaulieu and O'Meara 2016) on the 1000 bootstrap phylogenies trimmed to the *Cambaridae* subtree, including two character independent models, a full HiSSE model, and models in which only the cave or surface states had a hidden state. Taxa were coded as they were for the BiSSE analysis. The parameters of the “best‐fit” HiSSE model were used to reconstruct marginal ancestral states on the maximum‐likelihood phylogeny. All code used in our analyses is available in the Supporting Information.

## Results

### PHYLOGENY ESTIMATION AND SYNTHESIS

**Figure 1:**
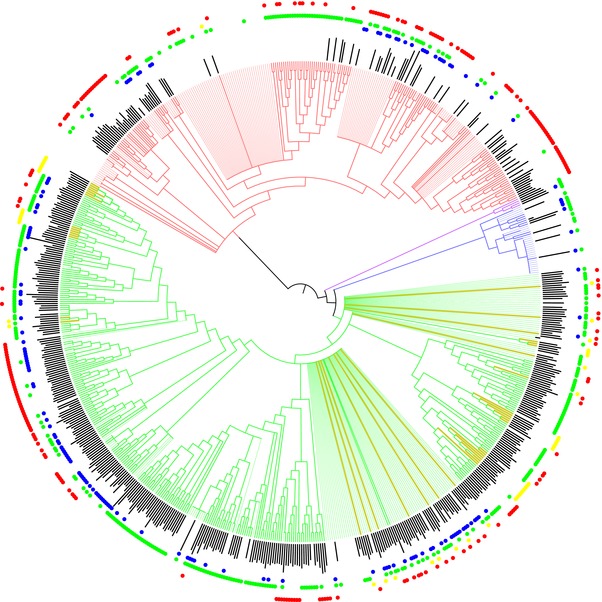
Phylogenetic synthesis of 19 studies, taxonomy, and the molecular phylogeny new to this study. Yellow branches lead to cave‐adapted taxa. Branches are colored by family: green, *Cambaridae*; blue, *Astacidae*; purple, *Cambaroididae*; red, *Parastacidae*. Solid branches lead to tips with molecular sequence data. Thin branches lead to tips represented only by Open Tree taxonomy. The black bars are values of log_10_ geographic range sizes in square kilometers. The outer ring of colored circles refers to habitat preferences: blue, lentic; green, lotic; yellow, cave; red, burrow. Figure was created using iTOL version 3 (Letunic and Bork 2016).

The maximum likelihood topology and divergence dates are largely concordant with other crayfish phylogenetics studies (Breinholt et al. 2012; Pedraza‐Lara et al. 2012; Ainscough et al. 2013; Bracken‐Grissom et al. 2014; Owen et al. 2015), but with the additional resolution of 119 taxa and 14 cave‐adapted species (Fig. S1; Dataset S3). The phylogeny synthesizing 19 phylogenetic studies, taxonomy, and our maximum likelihood molecular phylogeny contains 733 tips (including several undescribed species included in the OTT taxonomy). Of these, 466 have molecular phylogenetic data, 599 have habitat assignments, and 500 have range size data (Fig. 1; Tables S1 and S2). This phylogeny suggests up to 11 independent origins of cave adaptation although there still remain several described cave‐adapted species without molecular data. The concatenated alignment, best‐fit partitioning scheme determined by PartitionFinder, synthetic phylogeny, maximum likelihood phylogeny, and calibrated bootstrap trees are available in the Supporting Information.

### RANGE–HABITAT CORRELATION

Median range sizes were found to be highest in generalist species and lowest in cave dwellers (Table 1). All five of our threshold model analyses found significant correlations between liabilities in habitat preference and range size; namely, that lineages tend to have smaller range sizes when they are exclusively cave dwellers, primary burrowers, or lotic or lentic water inhabitants (Table 1). Conversely, lineages that occupy more than one habitat type tend to have larger range sizes (Table 1). The small range sizes found in cave lineages is in support of the evolutionary dead‐end hypothesis; however we do find small relative range sizes in all specialized lineages.

**Table 1:** Median geographic range sizes and liability correlation coefficients from threshold model analyses for each habitat type

Habitat preference	Median range size (km^2^)	Correlation coefficient
Lentic (*N* = 18)	19,777.37	−0.320 [−0.598:−0.013]
		(ESS = 291.13)
Lotic (*N* = 163)	30,481.20	−0.267 [−0.434:−0.080]
		(ESS = 1412.76)
Cave (*N* = 32)	16,323.35	−0.396 [−0.608:−0.168]
		(ESS = 298.84)
Primary burrower (*N* = 88)	23,580.75	−0.280 [−0.566:−0.017]
		(ESS = 322.50)
Generalist (*N* = 84)	69,753.43	0.604 [0.464:0.733]
		(ESS = 660.71)

Brackets contain the 95% HPD interval.

### HABITAT‐DEPENDENT DIVERSIFICATION

#### SSE model adequacy

The mean counts of tips in each state across the simulated trees were not significantly different from the empirical values for the GeoSSE and BiSSE analyses on the full tree and subtree (chi‐square, *P* > 0.1), indicating that these models adequately describe the diversification dynamics of this group using this metric. When considering the phylogenetic signal of these traits, only the GeoSSE analysis on the subtree produced simulated SSCD values that were not significantly different from the empirical values (*t*‐test, *P* > 0.25). Although the BiSSE analysis on the subtree did not pass this test (*P* < 0.01), 47.9% of empirical SSCD values did fall within the 50% distribution of the simulated SSCD values and 100% fell within in the 95% distribution. Backward simulations using GeoSSE and BiSSE parameters estimated on the full phylogeny failed to coalesce, suggesting that those estimates may be unrealistic. This is likely due to the heterogeneous diversification processes experienced across the group as a whole, particularly different patterns found in the Southern and North Hemisphere clades (Bracken‐Grissom et al. 2014; Owen et al. 2015), which violates these models.

Akaike information criterion (AIC) weights of HiSSE models supported a character‐dependent diversification model over an equally complex “character‐independent” model (Table S3). This suggests that using a “state‐dependent” diversification model in this analysis is appropriate. The HiSSE model with the highest AICw support was one with a “hidden” surface state (Table S3), suggesting that there are heterogeneous patterns of diversification in surface lineages and that diversification patterns across different cave clades are similar.

#### SSE model parameter estimates

**Figure 2:**
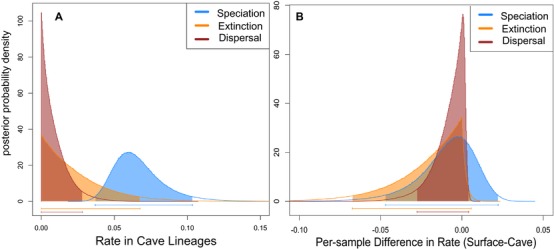
(A) Marginal posterior probability distributions of GeoSSE model parameters estimated for cave lineages from 1000 bootstrap phylogenies. (B) Per sample differences in rate estimates between surface and cave lineages.

As only the GeoSSE analysis on the subtree that minimized the tip‐state imbalance while still retaining a majority of taxa passed all of our model adequacy tests, we report only on those results here. We do note, however, that the BiSSE and GeoSSE analyses on the full phylogeny did not produce qualitatively different results. We found support for a “reversible” model of cave‐adaptation over an “irreversible” one (2lnBF = 4.278), suggesting that the dispersal rate out of caves is significantly nonzero, which is not in support of the evolutionary dead‐end model. However, the dispersal rate out of caves was less than both the speciation rate (posterior probability 0.99; Fig. 2A) and extinction rate (posterior probability 0.74; Fig. 2A), suggesting dispersal out of caves is quite rare. In fact, the speciation rate estimate was 72.6 times higher than the dispersal rate and the extinction rate was 26.0 times higher than the dispersal rate on average. Additionally, we did not find a significant difference between the dispersal rate into and out of caves (Fig. 2B), that is, the 95% HPD interval of the per‐sample difference between *d_C_* and *d_S_* includes 0. We found this same pattern in the speciation and extinction rates (Fig. 2B), suggesting that there are no significant differences in diversification patterns between cave and surface lineages, which does not support the evolutionary dead‐end model. The posterior probabilities that the extinction rate is greater than the speciation rate and speciation plus dispersal rate in cave lineages, as predicted by the evolutionary dead‐end model, were 0.002 and 0.001, respectively. On average, the estimated speciation rate was 3.1 times higher than the extinction rate.

**Figure 3:**
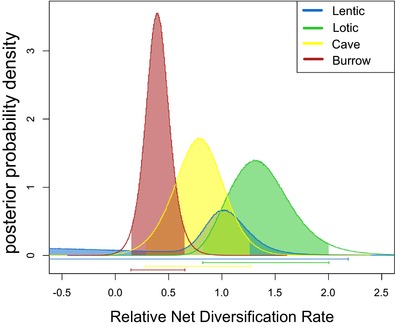
Relative net diversification rates estimated from separate GeoSSE analyses for each habitat type over 1000 bootstrap phylogenies.

Considering the four crayfish habitat categories, there was considerable overlap in the 95% HPD interval of relative net diversification rates ([*s_A_* − *x_A_*]/[*s_B_* − *x_B_*]; Fig. 3) with estimates in cave lineages overlapping each of the other three habitats, which is not in support of the evolutionary dead‐end hypothesis. The one outlier is the broad distribution in the relative net diversification rate estimate of “lentic” habitat dwellers. However, this may be an artifact due to the fact that only 20 species in the phylogeny were coded as exclusively lentic (Gamisch 2016). Although there was overlap in the distributions of these parameter estimates, the marginal posterior means were somewhat different across habitat states with no overlap between “lotic” habitat dwellers and primary burrowers (Fig. 3). This demonstrates that we were able to detect differences in rates among different habitat affinities and could explain why HiSSE favored a state‐dependent model over character‐independent ones and recovered multiple diversification schemes within the “surface” state (Table S3).

## Discussion

Karst (limestone) topography occupies 15% of the earth's surface (White et al. 1995), and despite critical and illuminating work from many speleobiologists over the years, caves are still a largely unexplored and poorly understood ecosystem (Culver and Pipan 2009; Perez‐Moreno et al. 2016). Subterranean organisms are frequently viewed as “oddities,” but perhaps this perception is guided by human inability to easily interact with the entirety of these ecosystems. Subterranean habitats have a cosmopolitan distribution with organismal exemplars across widely diverse taxonomic groups (Culver and Pipan 2009). Within the freshwater crayfish (some of the most conspicuous members of subterranean ecosystems they inhabit [Reynolds et al. 2013]), we find that there have been up to 11 transitions into caves (Figs. 1 and S1), but transitions from caves to epigean (surface) habitats are rare (Fig. 2). This is consistent with a pattern observed across cave organisms and is one of the reasons biologists have hypothesized caves to be “evolutionary dead‐ends” (Culver 1982; Culver and Wilkens 2000; Culver and Pipan 2009; Ribera et al. 2010; Niemiller et al. 2013). We find that rather than being an “evolutionary dead‐end,” cave‐adapted freshwater crayfish exhibit lineage diversification patterns that are nearly indistinguishable from their surface counterparts (Figs. 2 and 3).

We find that cave‐adapted lineages tend to have smaller geographic range sizes than surface lineages and this pattern is also found in other specialized habitat types when compared in a binary fashion (Table 1). On average, cave‐adapted crayfish have the smallest geographic range sizes of crayfish habitat types. This is expected due to the restricted dispersal ability of cave organisms and the fact that some cave crayfish are known from only one or a few localities (Barr and Holsinger 1985). Nevertheless, we find that the speciation rate in cave lineages is higher than both the extinction and transition rate (Fig. 2A). This is in agreement with other studies that cave crayfish are still able to disperse across subterranean landscapes and speciation can occur by subsequent restriction of gene flow (Buhay and Crandall 2005; Finlay et al. 2006). The limited dispersal ability we find suggests that range expansion occurs slowly and formation of new populations occurs rarely, but there is still enough dispersal ability for speciation to occur through evolutionary time. One might expect that with restricted ranges, susceptibility to environmental perturbations would increase extinction probability, but also increase speciation rates through population isolation (Barnosky 2001; Birand et al. 2012). We do not find evidence for increased speciation and extinction rates relative to surface lineages, suggesting that caves are as evolutionarily stable as other habitat types for freshwater crayfish (Figs. 2B and 3).

The term “evolutionary dead‐end” has been used to describe a number of different evolutionary patterns related to irreversibility (Schneider and Michalik 2011) or increased extinction rate (Agnarsson et al. 2006; Helanterä et al. 2009). It is often applied to specialists, depending on the situation, and holds a connotation of evolutionary failure (Wiegmann et al. 1993; Day et al. 2016). However, failure to diversify or transition back to an ancestral state does not signify a lack of success per se. Consider boreal conifers, which comprise only 0.3% of extant land plant diversity, have experienced low rates of lineage diversification but have large, stable populations with circumpolar distributions (Plomion et al. 2011). Cetaceans have never transitioned back to living on dry land, but are a large group of animals that have experienced high diversification rates (Steeman et al. 2009). We see similar patterns in cave crayfish. The uniquely dark, cold cave habitat results in its fauna developing certain morphologies, which may make them unsuccessful in other habitats, for example, loss of eyes and pigmentation. Despite the low‐energy input, lineages that are successful in colonizing this extreme habitat have the ability to thrive and spread across the subsurface range (Romero and Green 2005). Rather than a dead‐end, caves are a habitat with open niches available to those with the ability to colonize. Although troglobites are often considered examples of extreme specialization, one needs to consider the perspective with which we view these organisms. They may be specialized in that they are limited to one type of “habitat,” but if they are able to use a wide breadth of resources and niche‐spaces available to them, are they truly specialized (Futuyma and Moreno 1988)? Particularly when one considers the vast availability of subterranean habitats across the globe, it certainly does not seem to be the case.

Associate Editor: G. Thomas

Handling Editor: P. Tiffin

## Supporting information


**Dataset S1**. Concatenated alignment of six genes used for phylogenetic analysis along with best‐fit partitioning scheme.

**Dataset S2**. Synthetic tree file.

**Dataset S3**. Time‐calibrated, maximum‐likelihood molecular phylogeny.

**Dataset S4**. Time‐calibrated bootstrap phylogenies (*N* = 1000).

**Figure S1**. Marginal ancestral states estimated with the best‐fit HiSSE model on the maximum‐likelihood phylogeny trimmed to the *Cambaridae* subtree.

**File S1**. R scripts used to execute the state‐dependent diversification analyses and model adequacy tests.

**File S2**. Configuration file with fossil calibrations used in treePL.

**File S3**. Habitat state data used for GeoSSE analysis, derived from Table S2.

**File S4**. Habitat state data used for HiSSE analysis, derived from Table S2.

**Table S1**. Genbank accession numbers for all genes used in molecular phylogenetic analysis.

**Table S2**. Habitat assignments and log_10_ range size for taxa in the synthetic tree. Taxa without any data are excluded.

**Table S3**. AIC support for different models tested in HiSSE framework testing applicability of state‐dependent models. ΔAIC and AICw were calculated for each bootstrap phylogeny.
