# Supplementary figures and images for: Phylogenetic evidence from freshwater crayfishes that cave adaptation is not an evolutionary dead‐end
Source: Evolution. 2017 Sep 20;71(10):2522–32. doi: 10.1111/evo.13326 (PMC5656817; doi:10.1111/evo.13326)

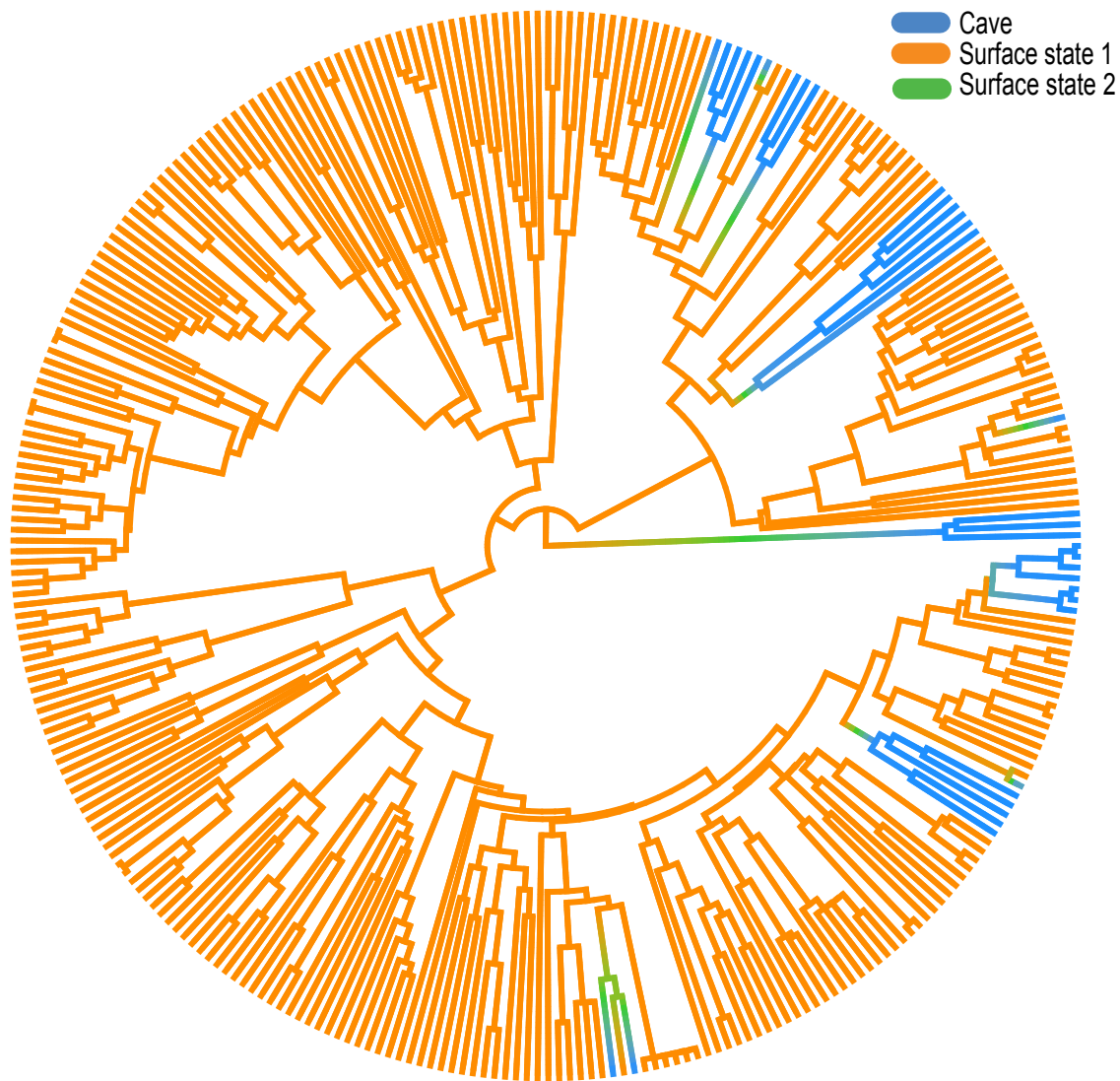

Supplement: Supplementary file 5 — Figure S1. Marginal ancestral states estimated with the best‐fit HiSSE model on the maximum‐likelihood phylogeny trimmed to the Cambaridae subtree. [file EVO-71-2522-s005.pdf]
